# Geographical Differentiation of Hom Mali Rice Cultivated in Different Regions of Thailand Using FTIR-ATR and NIR Spectroscopy

**DOI:** 10.3390/foods10081951

**Published:** 2021-08-22

**Authors:** Wannee Srinuttrakul, Alina Mihailova, Marivil D. Islam, Beatrix Liebisch, Florence Maxwell, Simon D. Kelly, Andrew Cannavan

**Affiliations:** 1 Research and Development Division, Thailand Institute of Nuclear Technology, Sai Mun, Ongkharak, Nakhon Nayok 26120, Thailand; wannees@tint.or.th; 2 Food and Environmental Protection Laboratory, Joint FAO/IAEA Centre of Nuclear Techniques in Food and Agriculture, Department of Nuclear Sciences and Applications, International Atomic Energy Agency, Vienna International Centre, P.O. Box 100, 1400 Vienna, Austria; M.Islam@iaea.org (M.D.I.); B.Liebisch@iaea.org (B.L.); F.Maxwell@iaea.org (F.M.); S.Kelly@iaea.org (S.D.K.); A.Cannavan@iaea.org (A.C.)

**Keywords:** Thai Hom Mali rice, geographical origin, authenticity, spectroscopy, FTIR-ATR, NIR

## Abstract

Although Hom Mali rice is considered the highest quality rice in Thailand, it is susceptible to adulteration and substitution. There is a need for rapid, low-cost and efficient analytical techniques for monitoring the authenticity and geographical origin of Thai Hom Mali rice. In this study, two infrared spectroscopy techniques, Fourier-transform infrared spectroscopy with attenuated total reflection (FTIR-ATR) and near-infrared (NIR) spectroscopy, were applied and compared for the differentiation of Thai Hom Mali rice from two geographical regions over two production years. The Orthogonal Projections to Latent Structures Discriminant Analysis (OPLS-DA) model, built using spectral data from the benchtop FTIR-ATR, achieved 96.97% and 100% correct classification of the test dataset for each of the production years, respectively. The OPLS-DA model, built using spectral data from the portable handheld NIR, achieved 84.85% and 86.96% correct classification of the test dataset for each of the production years, respectively. Direct NIR analysis of the polished rice grains (i.e., no sample preparation) was determined as reliable for analysis of ground rice samples. FTIR-ATR and NIR spectroscopic analysis both have significant potential as screening tools for the rapid detection of fraud issues related to the geographical origin of Thai Hom Mali rice.

## 1. Introduction

Rice is a staple food for over half of the world’s population and plays a major role in global food security [1]. In Thailand, rice is the most economically important crop. Thai Hom Mali rice, also known as Thai Jasmine rice, is a non-glutinous fragrant rice, considered the highest quality rice in Thailand and valued for its superb cooking quality, soft texture and aroma [2].

The unique aroma of Thai Hom Mali rice is linked to the content of 2-acetyl-1-pyrroline (2AP), which has been reported to be influenced by the production region [3]. The lowland rainfed area in the northeastern region of Thailand is an optimal environment for the accumulation of 2AP in rice grains [2,3]. It is widely acknowledged that Thai Hom Mali rice originating from the northeastern region of Thailand has superior organoleptic properties and is of higher quality. This creates an incentive for unscrupulous producers and retailers to mislabel rice originating from other geographical regions and represent it as more valuable rice cultivated in the northeast for financial gain [4]. These activities often remain undetected due to the lack of rapid analytical techniques that can be applied to screen samples in the supply chain.

A wide range of analytical approaches, including chromatography, mass spectrometry and genetic techniques, has been applied for the geographical traceability and authentication of rice [5]. The most widely applied techniques for the verification of geographical origin of rice are stable isotope analysis by isotope ratio mass spectrometry [6,7,8] and elemental analysis by inductively coupled plasma mass spectrometry [9,10,11]; often, these two techniques are combined to improve the discriminative power of an analysis [4,12,13,14,15,16]. Although the above methods have a strong theoretical basis linking rice characteristics to climate and soil geochemistry, their analysis, equipment and maintenance are somewhat sophisticated and costly. In addition, the above techniques require relatively lengthy sample preparation, which introduces a substantial delay between sampling and the generation of results. More rapid and cost-effective methods, which can be applied to screen samples both in the laboratory and directly in the field, are required for the geographical traceability of rice.

Infrared (IR) spectroscopy is a rapid, non-destructive technique that requires little or no sample preparation and does not involve the use of chemicals or specialized laboratory facilities [17,18,19]. Several studies have shown the potential of infrared spectroscopy combined with chemometrics for the differentiation of geographical and botanical origin of various food products, e.g., honey [20], cereals [21,22], extra virgin olive oil [23], butter [24], apples [25] and coffee [26].

Several studies have applied Fourier-transform infrared spectroscopy (FTIR) and near-infrared (NIR) spectroscopy for the authentication of rice. Chen, Tan, and Lin [27] applied NIR spectroscopy and support vector data description (SVDD) for the authentication of black rice samples (*n* = 142). The optimized SVDD model achieved acceptable performance with specificity of 100% and a sensitivity of 94.2% on the independent test set. The authors concluded that SVDD combined with NIR is a feasible and effective approach for the verification of the authenticity of black rice. Le Nguyen Doan, Nguyen, Marini and Biancolillo [28] used NIR spectroscopy coupled with partial least squares-discriminant analysis (PLS-DA) and soft independent modelling of class analogies (SIMCA) for the analysis of authentic (*n* = 72) and adulterated (*n* = 128) rice samples from Vietnam. The PLS-DA approach provided satisfactory results with total classification rates of 82.6% and 82.4% for authentic and adulterated samples, respectively. NIR spectroscopy was also reported as successfully discriminating authentic (*n* = 20) and adulterated (*n* = 140) rice from China [29]. Sampaio, Castanho, Almeida, Oliveira and Brites [30] applied NIR spectroscopy with principal component analysis (PCA), PLS-DA and support vector machines (SVM) for the discrimination and classification of rice varieties (*Indica* and *Japonica*) grown in Portugal. The accuracy, cross-validation and prediction achieved by the SVM model were 97%, 93% and 91%, respectively. McGrath et al. [31] used a two-tiered approach of rapid screening using portable NIR technology supported by second-tier testing using mass spectrometry-based analysis to differentiate between high value Basmati rice varieties and their potential adulterants, rice varieties with Protected Geographical Indication status from China, various qualities of rice in Ghana and Vietnam as well as locally produced and imported rice in Ghana. The study concluded that this two-tier approach can provide a substantially increased caliber of testing through rapid screening outside of the laboratory with the reassurance of corroborating mass spectrometry-based laboratory analysis to support decision making.

A limited number of studies have been reported on the application of IR spectroscopy for the authentication of Thai Hom Mali rice. Attaviroj, Kasemsumran and Noomhorm [32] applied FT-NIR spectroscopy for the discrimination of paddy rice samples (*n* = 259) from five varieties: Khao Dawk Mali 105 (KDML 105), Pathum Thani 1, Suphan Buri 60, Chainat 1 and Pitsanulok 2 using SIMCA and PLS-DA methods. The most accurate PLS-DA model demonstrated 97% correct identification for the KDML 105 variety and 100% for the others. The study showed the feasibility of FT-NIR spectroscopy analysis as a non-destructive technique for the rapid identification of paddy rice varieties in routine quality assurance testing. A recent study by Wongsaipun, Theanjumpol and Kittiwachana [33] analyzed authentic (*n* = 39) and adulterated (*n* = 423) Thai Jasmine rice samples using NIR spectroscopy and PLS regression. The chemometric model developed allowed the quantitative prediction of the adulterations in the rice. To our knowledge, there have been no studies addressing the regional differentiation of Thai Hom Mali rice using IR spectroscopy. In this study, we applied and compared two IR spectroscopy techniques, a benchtop FTIR-ATR and a low cost (<4000 USD), pocket size, handheld NIR, coupled with Orthogonal Projections to Latent Structures Discriminant Analysis (OPLS-DA), for the differentiation of Thai Hom Mali rice from the northeastern and northern regions. In addition, the effect of the sample preparation technique (grinding vs. no sample preparation) on the discriminative power of the OPLS-DA model, obtained with NIR spectral data, was assessed. We discuss the suitability of both spectroscopic techniques as tools for rapid testing of the geographical origin of Thai Hom Mali rice.

## 2. Materials and Methods

### 2.1. Samples

In total, 170 Thai Hom Mali rice samples were obtained for this study, collected from the northern and northeastern regions of Thailand during two production years: 2018 and 2019. In 2018, rice samples from the northeast were collected from the lower and central provinces, while in 2019 samples were collected from the upper and central provinces (Table 1).

Approximately 7 g of dehulled polished rice samples was ground to a fine powder using a kitchen grinder (1000 W, Moulinex la Moulinette, Écully, France). Whole rice grains and ground samples were stored in airtight containers at room temperature prior to analysis.

### 2.2. FTIR-ATR Analysis

FTIR spectra were collected between 4000 and 450 cm^−1^ at a resolution of 1 cm^−1^ using a benchtop FTIR spectrometer fitted with an attenuated total reflectance (ATR) accessory (Spectrum 2, Perkin Elmer, Beaconsfield, UK). Approximately 40 mg of a ground rice sample was placed on the ATR crystal and the powder was compressed with a consistent force (100 N) to achieve optimum transmission. Six scans were obtained for each sample. Spectra were converted to absorbance units prior to further processing.

### 2.3. NIR Analysis

NIR spectra were collected between 740 and 1070 nm at a resolution of 1 nm using a handheld NIR spectrometer (SCiO™, Consumer Physics, Tel-Aviv, Israel). Samples of ground rice (5 g) were dispensed into a glass Petri dish to a depth of approximately 3 mm. Samples were evenly distributed and scanned through the glass from underneath the Petri dish. Each sample was scanned in triplicate by a clockwise rotation of the Petri dish at 120° between the scans to ensure the average scan data would be representative of the larger sample. In addition to the ground samples, rice samples from 2019 (*n* = 100) were analyzed as whole grains. Whole dehulled polished rice grain samples (10 g) were placed into glass Petri dishes and scanned in triplicate as described above for the ground rice samples.

### 2.4. Data Pre-Processing and Statistical Analysis

Spectral data pre-processing and multivariate statistical analysis were performed using SIMCA multivariate data analysis software (Sartorius Data Analytics, Malmö, Sweden). The average of replicate scans was pre-processed using multiplicative scatter correction (MSC) and the 1st derivative functions.

The full dataset from each production year was divided in randomized order into the training dataset (*n* = 47 and *n* = 67 in years 2018 and 2019, respectively) and the test dataset (*n* = 23 and *n* = 33 in years 2018 and 2019, respectively). OPLS-DA with seven-fold cross-validation was used to build the discriminative models for the differentiation of northern and northeastern region in the training dataset. The performance of the models was assessed using the goodness of fit (R2) and predictability (Q2) values. Further, the obtained OPLS-DA models were used to predict the geographical origin of the samples from the test dataset. The predictive ability of the models was assessed using the correct classification rate of samples from each region. Further, the OPLS-DA model, obtained using the 2019 dataset (largest dataset), was used to predict the samples from the northern region (the only region with common provinces in both years) from the 2018 dataset. In addition, the effect of sample preparation (ground samples vs. no sample preparation) on the performance of OPLS-DA models, obtained using NIR spectral data, was assessed.

## 3. Results and Discussion

### 3.1. FTIR-ATR Spectroscopy

**Figure 1:**
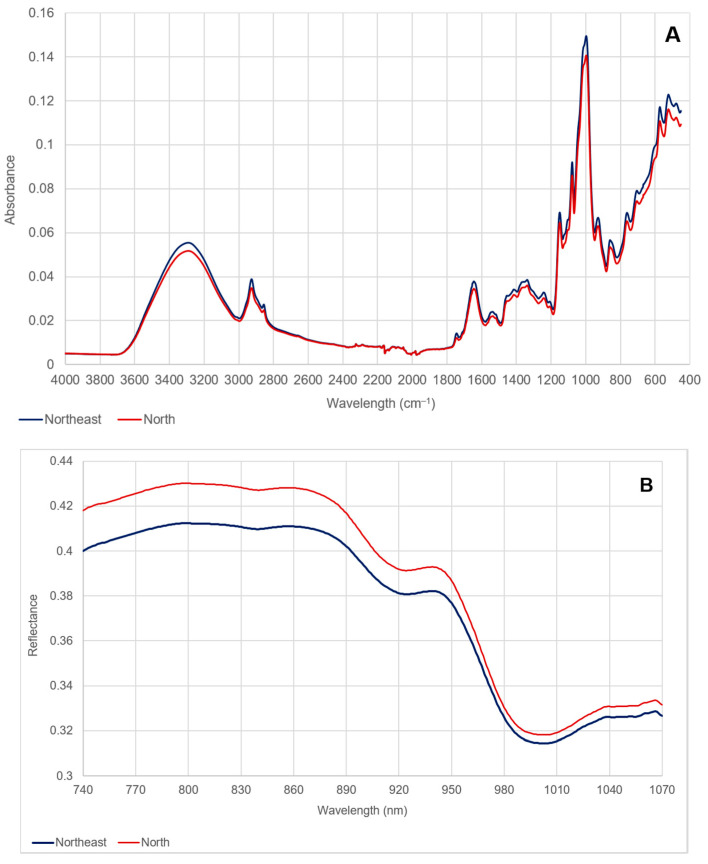
Mean raw FTIR-ATR (**A**) and NIR (**B**) spectra of Thai Hom Mali rice from the northern and northeastern regions (year 2019). Spectra from 2018 are similar and are not shown.

Representative FTIR absorbance spectra (4000 to 450 cm^−1^) obtained from Thai Hom Mali rice originating from the northern and northeastern regions are displayed in Figure 1A. The major differences in absorbance bands between the samples from the northeastern and northern regions were observed at approximately: 1030 and 1100 cm^−1^ (C-O stretching), associated with the presence of polysaccharides; 1540 cm^−1^ (N-H bend, C-N stretch) and 1640 cm^−1^ (C=O stretch), associated with the presence of proteins; and 2930 cm^−1^ (CH_2_ symmetric stretch), associated with the presence of lipids.

The obtained results agree with the earlier studies reporting the effect of season and production area on the chemical composition of Hom Mali rice [2]. Environmental variation in the cultivation regions affects the nitrogen, lipid, starch and fiber of polished rice [34]. Low-amylose rice such as Hom Mali has been reported to be particularly sensitive to environmental factors [35]. The northeastern region, which produces Hom Mali rice of the highest quality, differs from the northern region in terms of the amount of precipitation, elevation, soil type and fertility. The northern region is characterized by significantly higher elevation, annual precipitation and humidity. In contrast, the lowland rainfed area of the northeast has a cool, dry atmosphere during the ripening stage; sandy, mildly saline soil and poor fertility characterize this area, all of which affect both rice grain chemical composition and quality [2].

**Figure 2:**
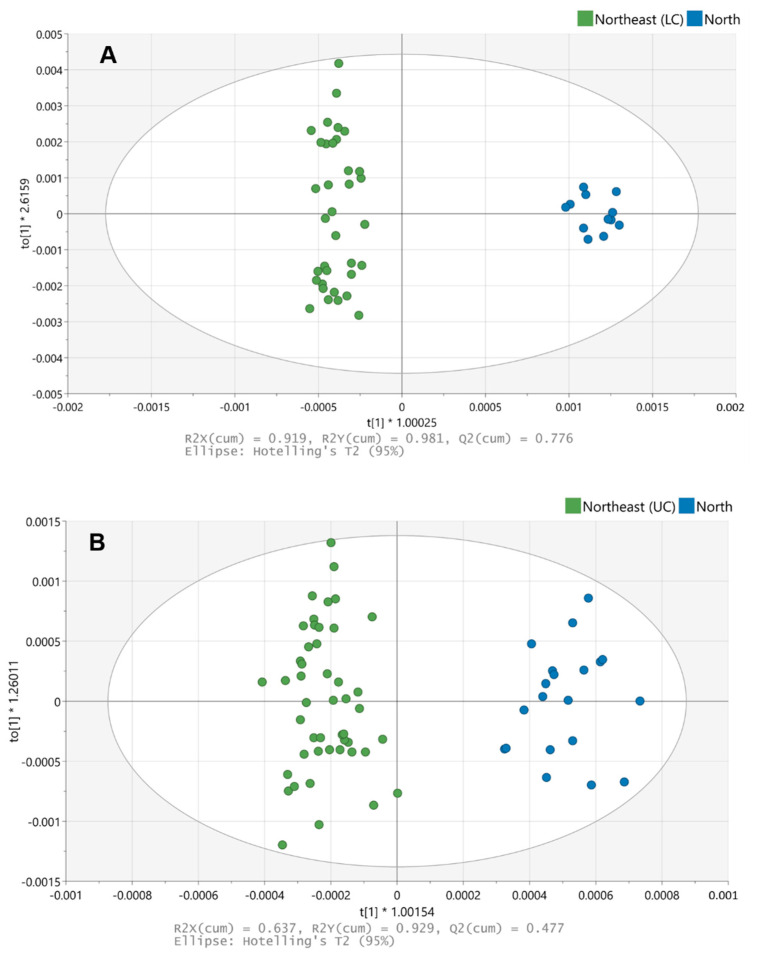
OPLS-DA models of the training dataset analyzed with FTIR-ATR: year 2018 (**A**) and 2019 (**B**).

OPLS-DA allowed for a clear differentiation between the rice samples from the northern and northeastern regions in both production years (Figure 2). The goodness of fit (R2) and the predictive ability (Q2) of the OPLS-DA model are shown in Table 2.

External model validation was performed using the test dataset comprising samples that were not used in the construction of the model. The correct classification rates of samples from the test dataset were 100% and 96.97% in 2018 and 2019, respectively.

Furthermore, the predictive ability of the OPLS-DA model, generated using the larger dataset of 2019, was challenged by using the samples of 2018 as “unknowns”. Because the samples from the northeastern region came from different provinces in 2018 and 2019, only samples from the north (common provinces in both years) were used for this additional model validation. The OPLS-DA model was able to correctly predict 100% of the “unknown” samples from the north from 2018 (Table 3).

### 3.2. NIR Spectroscopy

**Figure 3:**
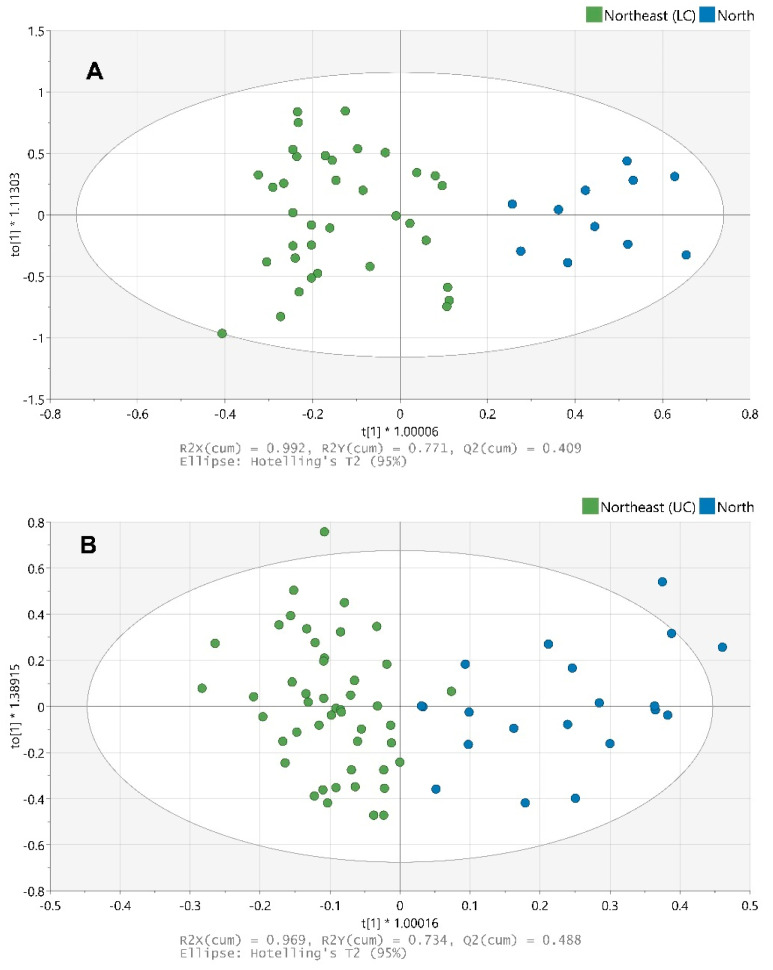
OPLS-DA models of the training dataset analyzed with NIR: year 2018 (**A**) and 2019 (**B**).

The NIR reflectance spectra (740 to 1070 nm) obtained from Thai Hom Mali rice originating from the northern and northeastern regions are displayed in Figure 1B. The NIR spectra, obtained using a handheld NIR spectrometer, allowed for the differentiation between rice samples from the northern and northeastern regions in both production years (Figure 3). The goodness of fit (R2) and predictive ability (Q2) of the OPLS-DA model are shown in Table 4. External model validation was performed using the test dataset comprising of samples that were not used in the construction of the model. The correct classification rates of samples from the test dataset were 86.96% and 84.85% in 2018 and 2019, respectively.

The predictive ability of the OPLS-DA model, generated using the larger dataset of 2019, was challenged by using the samples of 2018 as “unknowns”, as described in Section 3.1. The OPLS-DA model was able to predict 100% of the “unknown” samples from the north from 2018 (Table 3).

In addition to ground rice (*n* = 100), whole rice grain samples (*n* = 100) from 2019 were analyzed by handheld NIR, and the effect of both sample preparation techniques on the performance of OPLS-DA models was assessed. The results of the comparison of OPLS-DA models for ground and whole rice are shown in Table 4.

No decrease in model performance was observed for whole grain rice samples as compared to ground rice powder. The correct classification rates of samples from the test set were 87.88% and 84.85% for whole grain and ground rice samples, respectively. These results demonstrate that, in addition to its low cost, small size and ease of use, the handheld NIR spectrometer is suitable for the analysis of dehulled polished rice without the need for any sample preparation, which makes the approach rapid and straightforward and potentially applicable for point-of-use testing.

### 3.3. The Potential of FTIR and NIR Spectroscopy Techniques for Geographical Differentiation of Thai Hom Mali Rice

Stable isotope analysis, coupled with elemental analysis (SITE), has been successfully applied for the geographical differentiation of Thai Hom Mali rice [4]. However, these sophisticated analytical approaches require a substantial financial investment, skilled personnel and the use of chemicals, and thus cannot be applied outside the laboratory. In contrast, FTIR and NIR techniques are rapid, cost-effective, do not require specialized laboratory facilities and could therefore be used for pre-screening samples for SITE confirmatory or orthogonal analysis in enforcement work. Although FTIR and NIR spectroscopy were applied in several recent studies for the authentication of rice [27,28,29,30,31,33], to our knowledge, there have been no studies published that applied and compared benchtop FTIR-ATR and handheld NIR spectroscopy for differentiating the geographical origins of Thai Hom Mali rice.

The results of this study demonstrate the potential of the application of FTIR-ATR and NIR spectroscopy for the geographical differentiation of Thai Hom Mali rice. The performance values (R2 and Q2) of the OPLS-DA models and the correct classification rates of the samples from the test datasets (Table 2, Table 3 and Table 4) show that these spectroscopic techniques, coupled with chemometrics, can discriminate between the northeastern and northern regions and predict the origin of “unknown” samples from the test dataset.

The results of external model validation using the samples from the test dataset showed a lower correct classification rate for the handheld NIR spectrometer compared to the results obtained by the benchtop FTIR-ATR (Table 2 and Table 4). Considering the wavelength range, performance characteristics and physical dimensions (portability) of the handheld NIR, the lower performance of the OPLS-DA and lower correct classification rate of the test dataset is understandable. Advantages of the handheld NIR spectrometer include its low cost, portability and application outside the laboratory. Furthermore, the obtained spectral data are stored electronically in the “cloud” and can be accessed through a mobile app, allowing for handheld NIR devices to be used for cost-effective sample screening by which suspect fraudulent samples can be rapidly detected. The results obtained showed that no sample preparation was required for the handheld NIR spectrometer, thus allowing its use directly at a farm or retailer level and permitting for the early and more rapid detection of issues related to the authenticity of Thai Hom Mali rice.

Further studies using a larger number of authentic Hom Mali rice samples from a wider range of geographical locations in Thailand, as well as from other countries such as Cambodia and Vietnam, are required to build more robust classification and prediction models and to investigate their ability to reliably classify unknown samples.

## 4. Conclusions

Development of reliable, rapid non-targeted screening methods is important in identifying and preventing evolving fraudulent practices in the trade of Thai Hom Mali rice. This study has demonstrated that FTIR-ATR and NIR spectroscopy, combined with OPLS-DA, is a promising analytical tool for the geographical differentiation of Thai Hom Mali rice. Rice samples from the northeastern region were differentiated from the samples from the northern region in both production years. OPLS-DA models were validated with the samples from the test dataset, which were not used for the generation of the models. The OPLS-DA model built using spectral data from the benchtop FTIR-ATR achieved 96.97% and 100% correct classification of the test dataset for each of the production years, respectively. The OPLS-DA model built using spectral data from the handheld NIR achieved 84.85% and 86.96% correct classification of the test dataset for each of the production years, respectively.

Adapting and using these rapid screening techniques provides a promising way forward for the early detection of fraud at the farm and retail levels. The primary advantages of these techniques over traditional chemical and SITE methods are the speed and ease of use in routine operations, low cost, non-destructive nature of the techniques and minimal or zero (in case of handheld NIR) sample preparation. This approach allows for rapid pre-screening to identify suspect rice samples before committing to more sophisticated and time-consuming SITE techniques for confirmatory or orthogonal analysis. Further work using a wider range of authentic Hom Mali rice samples from multiple regions and production years is required to demonstrate the robustness of this approach.

## Figures and Tables

**Table 1:** Thai Hom Mali rice sample information.

Harvest Year	Region	Province	District	Number of Samples
2019	Northeast (UC)	Bueng Kan	Phon Charoen	5
2019	Northeast (UC)	Bueng Kan	Seka	5
2019	Northeast (UC)	Nakhon Phanom	Si Songkhram	5
2019	Northeast (UC)	Nakhon Phanom	That Phanom	5
2019	Northeast (UC)	Nong Khai	Phon Phisai	5
2019	Northeast (UC)	Nong Khai	Sakhrai	5
2019	Northeast (UC)	Sakon Nakhon	Phang Khon	10
2019	Northeast (UC)	Udon Thani	Kut Chap	10
2019	Northeast (UC)	Nong Bua Lam Phu	Na Klang	10
2019	Northeast (UC)	Khon Kaen	Khao Suan Kwang	10
2019	North	Phayao	Chun	10
2019	North	Phayao	Phu Sang	8
2019	North	Chiang Rai	Mae Chan	5
2019	North	Chiang Mai	Hang Dong	5
2019	North	Phayao	Chiang Muan	2
2018	North	Phayao	Phu Kamyao	4
2018	North	Phayao	Dok Khamtai	1
2018	North	Phayao	Chun	2
2018	North	Chiang Mai	Mae Rim	3
2018	North	Chiang Rai	Mae Chan	7
2018	Northeast (LC)	Kalasin	Mueang	10
2018	Northeast (LC)	Mukdahan	Khamcha-i	10
2018	Northeast (LC)	Yasothon	Mueang	5
2018	Northeast (LC)	Yasothon	Sai Mun	5
2018	Northeast (LC)	Roi Et	Kaset Wisai	5
2018	Northeast (LC)	Amnat Charoen	Mueang	3
2018	Northeast (LC)	Amnat Charoen	Senangkhanikhom	3
2018	Northeast (LC)	Ubon Ratchathani	Muang Sam Sip	3
2018	Northeast (LC)	Ubon Ratchathani	Det Udom	3
2018	Northeast (LC)	Surin	Chumphon Buri	3
2018	Northeast (LC)	Maha Sarakham	Phayakkhaphum Phisai	3

Northeast: UC—upper and central provinces; LC—lower and central provinces.

**Table 2:** Benchtop FTIR-ATR: goodness of fit and predictive ability of OPLS-DA models for the 2018 and 2019 datasets.

Year	N (Train. Set)	N (Test Set)	Model	R2X (cum)	R2Y (cum)	Q2 (cum)	Correct Classification Rate of the Test Set, %		
							Northeast	North	Total
2018	47	23	OPLS-DA	0.919	0.981	0.776	100	100	100
2019	67	33	OPLS-DA	0.637	0.929	0.477	96.65	100	96.97

N—number of samples; Train. Set—training set.

**Table 3:** Correct classification rate of the samples from the northern region obtained with OPLS-DA model using 2019 full dataset as a training set and 2018 full dataset as a test set.

Technique	N Train. Set (2019)	N Test Set (2018)	Model	R2X (cum)	R2Y (cum)	Q2 (cum)	Correct Classification Rate of the Test (North, *n* = 17), %
Benchtop FTIR-ATR	100	70	OPLS-DA	0.619	0.906	0.54	100
Handheld NIR	100	70	OPLS-DA	0.97	0.696	0.544	100

N—number of samples; Train. Set—training set.

**Table 4:** Handheld NIR: goodness of fit and predictive ability of OPLS-DA models for the 2018 and 2019 datasets.

Year	Rice Samples	N (Train. Set)	N (Test Set)	Model	R2X (cum)	R2Y (cum)	Q2 (cum)	Correct Classification Rate of the Test Set, %		
								Northeast	North	Total
2018	Ground	47	23	OPLS-DA	0.992	0.771	0.409	94.12	66.67	86.96
2019	Ground	67	33	OPLS-DA	0.969	0.734	0.488	91.30	70.00	84.85
2019	Whole grains	67	33	OPLS-DA	0.972	0.752	0.489	95.65	70.00	87.88

N—number of samples; Train. Set—training set.

## Data Availability

No data available.
